# Species-Specific Molecular Detection of the Root Knot Nematode *Meloidogyne luci*

**DOI:** 10.3390/biology10080775

**Published:** 2021-08-14

**Authors:** Carla Maleita, Joana M. S. Cardoso, Leidy Rusinque, Ivânia Esteves, Isabel Abrantes

**Affiliations:** 1 Department of Chemical Engineering, Chemical Process Engineering and Forest Products Research Centre, University of Coimbra, Rua Sílvio Lima, Pólo II—Pinhal de Marrocos, 3030-790 Coimbra, Portugal; 2 Department of Life Sciences, Calçada Martim de Freitas, University of Coimbra, Centre of Functional Ecology, 3000-456 Coimbra, Portugal; joana.cardoso@uc.pt (J.M.S.C.); leidy.rusinque@iniav.pt (L.R.); iesteves@uc.pt (I.E.); isabel.abrantes@uc.pt (I.A.); 3 Instituto Nacional de Investigação Agrária e Veterinária (INIAV, I.P.), 2780-159 Oeiras, Portugal

**Keywords:** diagnosis, RAPD, species-specific primer, sequence characterized amplified region

## Abstract

**Simple Summary:**

The root knot nematode *Meloidogyne luci* has been identified in various countries around the world parasitizing economically important crops. Due to its potential to cause serious damage to agriculture, the need for an accurate diagnosis at the species level has become mandatory. In the present study, a specific amplification product on *M. luci* was obtained from a random amplified polymorphic DNA (RAPD) analysis. The DNA was sequenced and converted into a sequence characterized amplified region (SCAR) marker used for the species-specific molecular detection of this root knot nematode. The developed methodology is essential to monitoring the distribution and spread of *M. luci* in order to implement future effective and integrated pest management programs.

**Abstract:**

*Meloidogyne luci* has been identified in various countries around the world parasitizing economically important crops and, due to its potential to cause serious damage to agriculture, was included in the European and Mediterranean Plant Protection Organization Alert List in 2017. This species shares morphological and molecular similarities with *M. ethiopica* and *M. inornata*, and a *M. ethiopica* group was therefore established. Although specific primers for the DNA amplification of species belonging to the *M. ethiopica* group have been developed previously, the primers were not species-specific, so molecular markers for the specific detection of *M. luci* are still needed. The objective of this study was to develop a SCAR marker for the detection of *M. luci* and the discrimination from other *Meloidogyne* spp. based on the intraspecific variability found in RAPD markers. RAPD screening of *M. luci* and *M. ethiopica* genome was used for the identification of a specific amplification product on *M. luci*, which was cloned, sequenced and converted into a SCAR marker. The specificity of the designed primers (Mlf/r) was tested and produced a fragment (771 bp) for all nine *M. luci* isolates with no amplification for the other nine *Meloidogyne* spp., including *M. ethiopica* and *M. inornata*. Additionally, the proper amplification of the *M. luci* SCAR-marker was also successful with DNA from galls of *M. luci* infected tomato roots. The results obtained in this study reveal that the specific molecular detection of *M. luci* was achieved and that the developed methodology can be used for routine diagnosis purposes, which are essential to monitoring the distribution and spread of *M. luci* in order to implement future effective and integrated nematode pest management programs.

## 1. Introduction

Root knot nematodes (RKN), *Meloidogyne* spp., comprise one of the most successful groups of plant parasites, responsible for worldwide crop losses of billion dollars annually [1]. The impact of RKN in agricultural areas strengthens the need for an accurate diagnosis at the species level. At the moment, the genus *Meloidogyne* includes 105 described species of which four are considered the most common (*M. arenaria*, *M. hapla*, *M. incognita* and *M. javanica*) due to their wide distribution and host range, but many others have been recognized as emerging species [1,2,3]. For instance, *M. chitwoodi*, *M. enterolobii*, *M. fallax* and *M. mali* are included in the European and Mediterranean Plant Protection Organization (EPPO) A2 List of pests recommended for regulation as quarantine organisms, whereas *M. ethiopica, M. graminicola* and *M. luci*, based on their potential to cause serious damage to agriculture, were added to the EPPO Alert List [4].

In the past, RKN identification was frequently centered on microscopic examination and female perineal pattern analyses, but these methods are often unreliable and require specialized skills due to the inter- and intraspecific RKN variability and to the frequent occurrence of more than one species in the same sample [5,6]. Currently, the biochemical electrophoretic analysis of non-specific esterases is a widely used method used to differentiate *Meloidogyne* species, with many species-specific isozyme patterns already published [6]. However, the need for fully developed female nematodes in good condition, the observation of intraspecific isozyme phenotypic variability, the resemblance between species based on gel comparative analysis of esterase patterns, the discovery of new patterns, time-consuming methodology and the difficulty of processing a large number of samples are all constraints that complicate RKN biochemical identification. Thus, additional information provided by DNA-based methodologies are crucial in RKN diagnostics. Random amplified polymorphic DNA (RAPD), restriction fragment length polymorphism variation (RFLP) and sequence characterized amplified regions (SCAR) markers have been developed, and different DNA regions have been used for the identification and phylogenetic analysis of RKN [7,8,9,10,11,12,13,14,15,16].

The emerging RKN *M. luci*, obtained from lavender roots (*Lavandula spica* L.) in Brazil, was described in 2014 [17]. This species shares some morphological and biochemical similarities with *M. ethiopica* and *M. inornata,* which led to the initial misidentification of several *M. luci* isolates in Europe as *M. ethiopica* [15,16]. *Meloidogyne luci* has been identified in various countries around the world, namely Argentina, Brazil, Bolivia, Chile, Ecuador, Greece, Guatemala, Iran, Italy, Portugal, Slovenia and Turkey, and is associated with more than 40 economically important crops, ornamentals, herbs and weeds [15,17,18,19,20,21,22,23,24,25,26,27,28,29,30,31,32,33,34].

Important crop losses have been referred on tomato, with reports of more than 80% crop decline caused by *M. luci* in a greenhouse in Slovenia [26]. On potato, the pathogenicity of *M. luci* was studied in 16 commercial cultivars, and all the cultivars were susceptible to this nematode, possessing relatively high pest reproduction factors [27]. Recently, it was shown that *M. luci* can cause latent infestation without visible infestation symptoms on the surface of potato tubers or severe tuber infestation with visible infestation symptoms [34].

In the present study, a suitable and accurate molecular method for the detection of *M. luci* is presented, discriminating this species from the closely related *M. ethiopica* and *M. inornata*. The efficient and fast identification of *M. luci* is essential to monitoring its distribution and spread and to implementing future effective and integrated pest management programs.

## 2. Materials and Methods

### 2.1. Nematode Isolates

Nine *M. luci* isolates from different origins and hosts were used in this study. Nine isolates from other RKN species were also included for the comparison and validation of the species-specific detection (Table 1).

All of the isolates were selected from the collection of the NEMATO-lab (CFE-UC) and maintained on tomato (*Solanum lycopersicum* L., cv. Coração-de-Boi) by periodic sub-culturing in a growth chamber (~25 °C; 12 h photoperiod) by transferring the culture, every 2 months, to new tomato seedlings.

### 2.2. Nematode Species Identification

Before DNA extraction, the identification of all RKN isolates was confirmed by the biochemical electrophoretic analysis of the non-specific esterase phenotype of females, as previously described [27]. Five egg-laying females of each RKN isolate were handpicked from infected tomato roots and transferred to micro-hematocrit tubes with 5 µL of 20% sucrose and 1% Triton X-100. The females were macerated, frozen and stored at −20 °C. The electrophoresis was carried out using the Mini-Protean Tetra Cell System (Bio-Rad Laboratories). The polyacrylamide gels were stained for esterase activity with α-naphthyl acetate. Protein extracts from five females of *M. javanica* were included in each gel for reference.

### 2.3. DNA Extraction

Egg masses were handpicked from tomato cv. Coração-de-Boi infected roots, placed in a hatching chamber and kept in the dark (25 °C). Hatched second-stage juveniles (J2) were collected and stored at −20 °C. Total genomic DNA was extracted from J2 of each *Meloidogyne* isolate using the DNeasy Blood & Tissue Kit (QIAGEN). Genomic DNA was also extracted from galls of *M. luci* (isolate PtL1) and *M. ethiopica* (isolate BrEt) infected tomato roots. Single galls with single third-stage juvenile to young females without egg mass were handpicked, crushed with a piston, on ice, and then DNA was extracted using the DNeasy Plant Mini Kit (QIAGEN), according to the manufacturer’s instructions.

### 2.4. Random Amplified Polymorphic DNA (RAPD) Analysis

Twenty-one random 10-mer oligo-nucleotide primers (Table 2) were used for RAPD *M. luci* (isolate PtL1) and *M. ethiopica* (isolate BrEt) genome screening to find DNA markers specific to *M. luci*.

PCR reactions were performed in 13 µL volume containing 10 ng of *M. luci* or *M. ethiopica* DNA, 1× buffer, 1.8 mM MgCl_2_, 0.2 mM dNTPs, 0.3 µM of primer and 2 U BioTaq DNA polymerase (Bioline). The amplifications were carried out in a thermal cycler (Bio-Rad) using the following conditions: an initial denaturation at 94 °C for 5 min, followed by 40 cycles of denaturation at 94 °C for 30 s, annealing at 39 °C for 45 s and extension at 72 °C for 2 min, and a final extension for 10 min at 72 °C. The PCR products were analyzed on 1.5 % agarose gel electrophoresis in 1× TBE buffer stained with GreenSafe (Nzytech). The experiment was repeated twice to confirm the reproducibility of the results.

### 2.5. Cloning and Sequencing RAPD Fragment

A RAPD amplification product present on *M. luci* and absent on *M. ethiopica* was purified from the agarose gel using the NucleoSpin^®^ Gel and PCR Clean-up kit (Macherey Nagel), ligated into a pGEM^®^-T Easy Vector (Promega) and transformed in *Escherichia coli* JM109 High Efficiency Competent Cells (Promega), following the manufacturer’s instructions. Plasmid DNA was extracted from *E. coli* cells using a NZYMini Prep kit (Nzytech), and two positive clones were selected and were fully sequenced in both strands in an Automatic Sequencer 3730xl under BigDyeTM terminator cycling conditions at the Macrogen Company (Spain).

### 2.6. Primer Design and PCR for Sequence Characterized Amplified Region (SCAR)

Based on the obtained sequence, a pair of species-specific primers in the two extremes of the SCAR and spanning the OPY-11 RAPD primer sequence were designed using a Primer-BLAST tool [39]. These primers were then used in PCR. Amplifications were carried out using 50 ng of extracted DNA and 2 U of BioTaq DNA polymerase (Bioline) in 1× reaction buffer, 0.2 mM each dNTPs, 1.8 mM MgCl_2_ and 0.2 µM of each primer (Mlf 5′-ACTCCTGCGACCTCATGGCATTTA-3′ and Mlr 5′-ACTCCTGCGAACACAACATTTACT-3′). Reactions were carried out in a thermal cycler (Bio-Rad) with an initial denaturation step of 94 °C for 4 min followed by 35 reaction cycles of 94 °C for 30 s, then annealed for 45 s at 70 °C, with an extension at 72 °C for 45 s and a final extension at 72 °C for 10 min. The specificity of the designed primers was tested using DNA from nine *M. luci* isolates and one isolate of *M. arenaria*, *M. chitwoodi, M. enterolobii*, *M. ethiopica*, *M. hapla, M. hispanica*, *M. incognita, M. inornata* and *M. javanica* (Table 1).

Additionally, DNA from the galls of tomato roots infected with *M. luci* (isolate PtL1) or *M. ethiopica* (isolate BrEt) were amplified using the same amplification conditions of SCAR-PCR. A positive control was included (DNA from J2 of PtL1). To increase the sensitivity of the test, a second PCR was performed using the same PCR conditions as described above, with 1 µL of the first PCR reaction (or 1 µL of 1:10 dilution for positive control) used as template.

The amplification of the COI of the mtDNA region was also performed in order to confirm the success of nematode DNA extraction from the J2/galls of non-target samples using primers and PCR conditions described in [27] (data not shown).

All the PCR products were analyzed on 1% agarose gel electrophoresis in 1× TBE buffer stained with GreenSafe. The experiments were repeated with at least two biological replicates, with DNA extracted from two different samples for each isolate.

## 3. Results

### 3.1. Esterase Phenotypes

**Figure 1:**
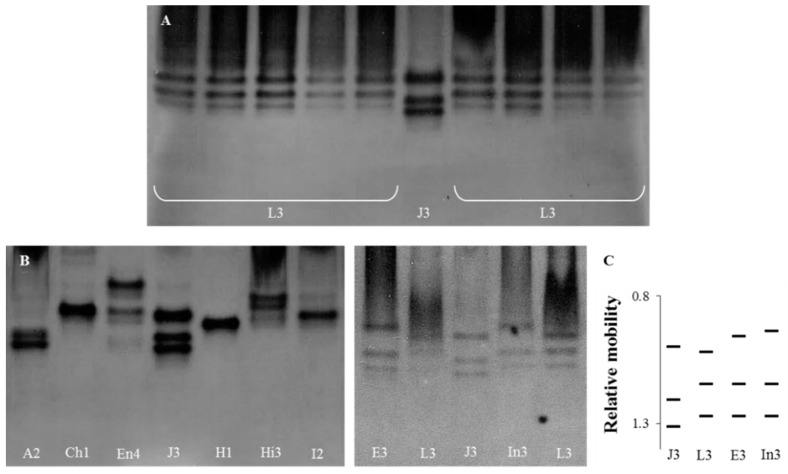
Esterase phenotypes of protein homogenates from five egg-laying females of *Meloidogyne* species isolates. From left to right: (**A**) L3—*M. luci* (isolates PtL1, PtL2, PtL3, BrL and GrL1), J3—*M. javanica* (reference isolate); L3—*M.* *luci* (isolates GrL2, ItL, SvL and TrL); (**B**) A2—*M. arenaria*; Ch1—*M. chitwoodi*; En4—*M. enterolobii*; J3—*M. javanica* (reference isolate); H1—*M. hapla*; Hi3—*M. hispanica*; I2—*M. incognita*; E3—*M. ethiopica*; L3—*M. luci* (PtL1); J3—*M. javanica* (reference isolate); and In3—*M. inornata*. (**C**) Relative mobility of J3, L3, E3 and In3. For isolate codes see Table 1.

The identification of all *Meloidogyne* isolates was confirmed by the esterase phenotypes (Figure 1). The three esterase bands (J3) observed in the *M. javanica* isolate were used as a reference phenotype, which allowed for the determination of the relative position of the bands perceived in the *Meloidogyne* isolates.

In the *M. luci* isolates, three bands of esterase activity were detected, corresponding to the phenotype L3, attributed to *M. luci* from Brazil (Figure 1A). The other nine *Meloidogyne* spp. isolates exhibited 22 bands of esterase activity, comprising nine phenotypes based on single bands or combinations. All the species displayed distinct and typical species-specific phenotypes, as previously described: *M. arenaria* (A2), *M. chitwoodi* (Ch1), *M. enterolobii* (En4), *M. ethiopica* (E3), *M. javanica* (J3), *M. hapla* (H1), *M. hispanica* (Hi3), *M. incognita* (I2) and *M. inornata* (In3) (Figure 1). The esterase phenotype of *M. luci* was distinct from those of *M. ethiopica* and *M. inornata* and from the other *Meloidogyne* spp. (Figure 1B).

### 3.2. Selection of Meloidogyne luci Specific RAPD Fragment

**Figure 2:**
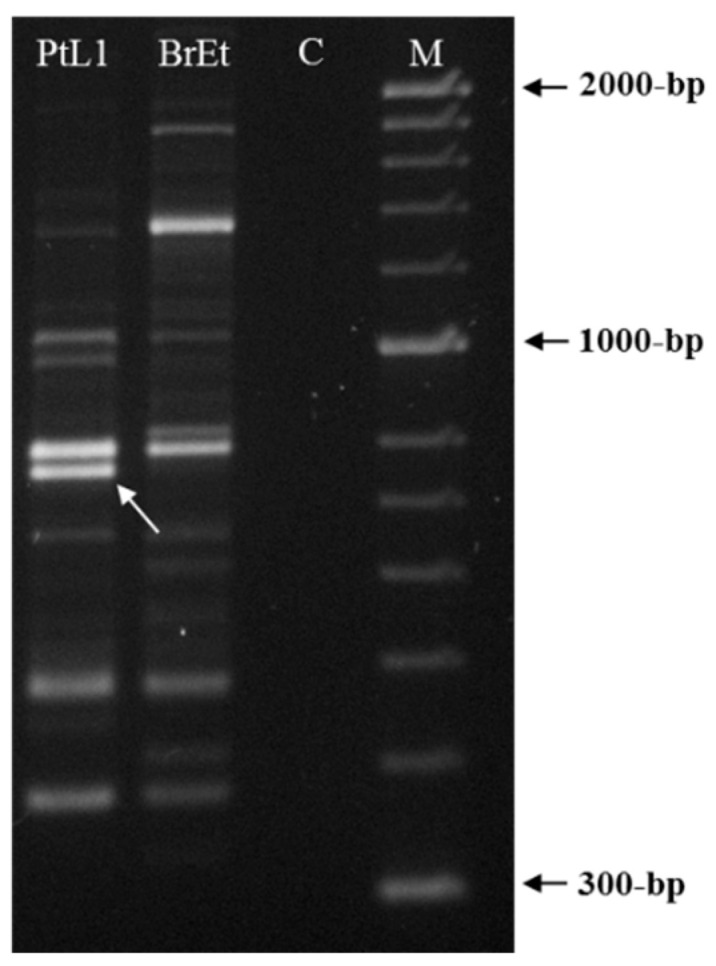
RAPD patterns for *Meloidogyne luci* (isolate PtL1) and *M. ethiopica* (isolate BrEt) obtained with primer OPY-11. The *M. luci* specific ~770 bp fragment is shown with an arrowhead. For isolate codes, see Table 1. C—Negative control; M—DNA marker (HyperLadder II, Bioline).

A total of 21 random 10-mer oligo-nucleotide primers were used to find a species-specific marker for *M. luci* (Table 2). Most primers were unable to differentiate *M. luci* from *M. ethiopica* or the results were not reproducible and intraspecific variability among *M. luci* isolates was also found (data not shown), except for primer OPY-11. The primer OPY-11 resulted in different band patterns for *M. luci* (isolate PtL1) and *M. ethiopica* (isolate BrEt) isolates (Figure 2). A consistent species-specific band of ~770-bp was present in the *M. luci* isolate and absent in the *M. ethiopica* isolate. This DNA fragment was cloned and the sequence was determined to be used as a *M. luci* SCAR marker.

### 3.3. Meloidogyne luci SCAR Marker and Species-Specific Detection Assay

**Figure 3:**
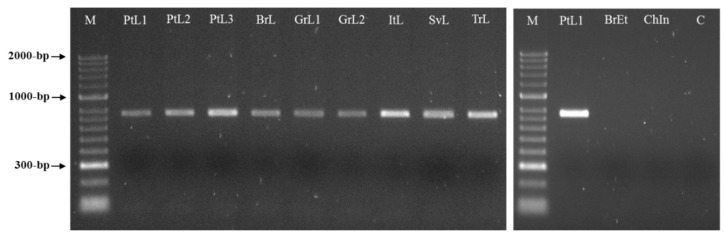
SCAR-PCR products (~770 bp specific fragment) for *Meloidogyne luci* (isolates PtL1, PtL2, PtL3, BrL, GrL1, GrL2, ItL, SvL and TrL), *M. ethiopica* (isolate BrEt) and *M. inornata* (isolate ChIn) using the species-specific primer pair Mlf/r. For isolate codes, see Table 1. M—DNA marker (HyperLadder II, Bioline); C—Negative control.

**Figure 4:**
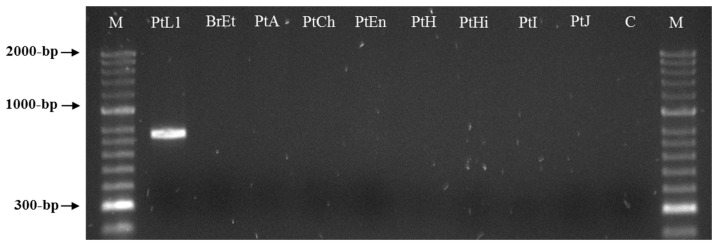
SCAR-PCR product (~770 bp specific fragment) for *Meloidogyne luci* (isolate PtL1) and other *Meloidogyne* species (isolates BrEt, PtA, PtCh, PtEn, PtH, PtHi, PtI and PtJ) included in the study, using the species-specific primer pair Mlf/r. For isolate codes see Table 1. M—DNA marker (HyperLadder II, Bioline); C—Negative control.

**Figure 5:**
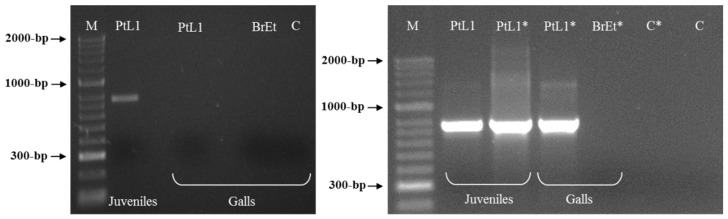
PCR product for *Meloidogyne luci* (isolate PtL1) infected tomato root (individual gall) using the species-specific primer pair Mlf/r. Tomato infected root with *M. ethiopica* (isolate BrEt) was included for comparison. For isolate codes see Table 1. * Re-amplification; M—DNA marker (HyperLadder II, Bioline); C—Negative control.

Two sequences of 771 bp were obtained by sequencing two clones of the selected RAPD fragment and submitted to GenBank under the accession numbers MW922841 and MW922842. Based on these sequences, the primers Mlf (5′-ACTCCTGCGACCTCATGGCATTTA-3′) and Mlr (5′-ACTCCTGCGAACACAACATTTACT-3′) were designed and used for the PCR amplification of the *M. luci* SCAR marker (771 bp). This was successfully amplified for all *M. luci* isolates with no amplification registered for the other *Meloidogyne* spp., namely, *M. arenaria*, *M. chitwoodi, M. enterolobii*, *M. ethiopica, M. hapla, M. hispanica*, *M. incognita, M. inornata* and *M. javanica* (Figure 3 and Figure 4). Additionally, the proper amplification of the *M. luci* SCAR-marker was also successfully achieved using DNA from the galls of *M. luci* infected tomato roots. On the other hand, no amplification was obtained with the DNA from the galls of the *M. ethiopica* infected tomato roots (Figure 5).

## 4. Discussion

*Meloidogyne luci* is a polyphagous species with a wide host range including plants from several botanical families. It affects important crops such as potato, tomato, maize, bean and kiwifruit, among others [24,27,28,29,30,40]. As *M. luci* has been included in the EPPO Alert List of harmful organisms since 2017, due to its increasing importance and potential to cause serious damage to agriculture [41], it is crucial to have rapid and discriminative methods for detecting the presence of this emerging RKN.

*Meloidogyne luci* and *M. ethiopica* are morphologically very similar, and their molecular relationship has been the subject of several studies ever since the *M. luci* description, with different DNA regions being characterized, namely: internal transcribed spacer 1 (ITS1) rRNA and D2-D3 fragment of 28S rRNA regions [17,25]; mitochondrial DNA (mtDNA) cytochrome oxidase subunit II (COII) [15,27]; and ITS1-5.8S-ITS2 rRNA region and cytochrome oxidase subunit I (COI) mtDNA region [27]. However, the ITS and D2-D3 fragments of the 28S rRNA region were considered inappropriate for studying the relationships among these closely related RKN species [15,27]. Gerič Stare et al. [15] selected the mtDNA COII region as the most useful for the identification and differentiation of *M. luci* from closely related species, whereas Maleita et al. [27] designated the mtDNA COI region as the most effective. Due to a lack of consensus about the true significance of the close relationship between *M. luci*, *M. ethiopica* and *M. inornata*, Gerič Stare et al. [16] proposed the integration of these three species in a separate group, forming the *M. ethiopica* group, based on a unique structure of *map-1* genes when compared to the other tropical RKN species. Furthermore, Gerič Stare et al. [16] developed a pair of primers, designed on COII mtDNA region, for the molecular detection of *M. ethiopica* group by PCR. The use of these primers was further validated for the detection of nematodes of the *M. ethiopica* group by real-time PCR, but they do not allow the discrimination of species within the group [34]. In order to obtain insight on the phylogeny of the RKN, Álvarez-Ortega et al. [42] used a multigene dataset (18S rRNA, ITS1 rRNA, D2-D3 expansion segments of 28S rRNA, COI gene and COII-16S rRNA) and incorporated the representatives of the *M. ethiopica* group in the same clade with other 17 RKN species distributed in warmer climates.

In the present study, a *M. luci* specific SCAR marker (771 bp) was obtained with the OPY-11 RAPD primer and somewhat similar sequences of this SCAR product were found in the recently published *M. luci* draft genome [43]. Based on this SCAR product, species-specific primers for *M. luci* were designed, providing the first diagnostic molecular method for the specific detection of *M. luci* and allowing for the discrimination of this species from the closely related *M. ethiopica* and *M. inornata* [16]. Although a SCAR marker has been previously developed for the specific detection of *M. ethiopica* [13], the discrimination/identification of the three species within the *M. ethiopica* group, until now, was only possible by the isoenzyme analysis. Nevertheless, the esterase phenotype assessment has limitations, as it requires young females and a gel comparative analysis of the obtained patterns for the different species. The first band of the esterase phenotype of *M. luci* is located at the same level of the first band of *M. javanica* (reference isolate), whereas the first bands of *M. ethiopica* and *M. inornata* are located above, making it almost impossible to distinguish them when they are not in the same gel [14,15,17,27,30,44,45].

## 5. Conclusions

The *M. luci* species-specific PCR based assay, described in this study, is accurate and highly sensitive since it requires minimal DNA templates from nematode eggs, juveniles, females or infected plant material for the amplification and detection of target sequences. The specificity of the developed primer pair Mlf/r was also validated by including *M. luci* isolates from different origins and hosts and other seven isolates from RKN species than *M. ethiopica* and *M. inornata*.

The application of a species-specific PCR-SCAR for the *M. luci* detection on the galls of infected tomato roots was also investigated, and our findings revealed that the molecular differentiation of *M. luci* in galls is reachable and that the methodology can be adopted in routine inspections or for monitoring distribution and spread of this emerging plant pathogen.

## Figures and Tables

**Table 1:** *Meloidogyne* isolates: hosts and geographic origin.

Species (Isolate Code) ^a^	Host Plant	Geographic Origin	Reference
*M. luci*(PtL1)	*Solanum tuberosum* L.	Coimbra	[27]
(PtL2)	*Oxalis corniculata* L.	Montemor-o-Velho	[30]
(PtL3)	*Cordyline australis* (Forst f.) Hook. F	Figueira da Foz	[30]
(BrL)	*Phaseolus vulgaris* L.	Paraná State	[25]
(GrL1)	*Zea mays* L.	Kavalla	[15,21]
(GrL2)	*Actinidia* sp.	Kavalla	[15,21]
(ItL)	*S. lycopersicum* L.	Pontecagnano	[15,22]
(SvL)	*S. lycopersicum* L.	Dornberk	[15,18]
(TrL)	*Cucumis sativus* L.	Çarsamba	[15,23]
*M. arenaria* (PtA)	*Crassula multicava* Lem.	Coimbra	—
*M. chitwoodi* (PtCh)	*S. tuberosum* L.	Porto	[35]
*M. enterolobii* (PtEn)	*Cereus hildmannianus* K. Schum.	Montemor-o-Velho	[36]
*M. ethiopica* (BrEt)	*Actinidia deliciosa* (Chevalier) Liang & Ferguson	Rio Grande do Sul State	[37]
*M. hapla* (PtHa)	*S. lycopersicum* L.	Montemor-o-Velho	—
*M. hispanica* (PtHi)	*Ficus carica* L.	Setúbal	[38]
*M. incognita* (PtI)	*Cucumis melo* L.	Azores	[22]
*M. inornata* (ChIn) ^b^	*S. lycopersicum* L.	Chile	—
*M. javanica* (PtJ)	*S. tuberosum* L.	Guarda	[22]

^a^ Br—Brazil; Ch—Chile; Gr—Greece; It—Italy; Pt—Portugal; Sv—Slovenia; Tr—Turkey. ^b^ Original population number—NPPO-NL F2642.

**Table 2:** Random amplified polymorphic DNA primers used in this study.

Primer Name	Primer Sequence (5′→3′)
OPA-06	GGTCCCTGAC
OPA-08	GTGACGTAGG
OPA-09	GGGTAACGCC
OPA-17	GACCGCTTGT
OPAB-05	CCCGAAGCGA
OPAS-09	TGGAGTCCCC
OPB-01	GTTTCGCTCC
OPB-14	TCCGCTCTGG
OPC-06	GAACGGACTC
OPC-08	TGGACCGGTG
OPD-01	ACCGCGAAGG
OPE-06	AAGACCCCTC
OPE-07	AGATGCAGCC
OPF-07	CCGATATCCC
OPG-04	AGCGTGTCTG
OPK-02	GTCTCCGCAA
OPM-01	GTTGGTGGCT
OPN-11	CTCACGTTGG
OPO-06	CCACGGGAAG
OPR-09	TGAGCACGAG
OPY-11	AGACGATGGG

## Data Availability

Sequence data that support part of the findings of this study are available in GenBank under the accession numbers MW922841 and MW922842. Other data generated during this study are included in this article.
